# Species Turnover and Diel Flight Activity of Species of Dung Beetles, *Onthophagus*, in the Tropical Lowland Forest of Peninsular Thailand

**DOI:** 10.1673/031.012.7701

**Published:** 2012-07-05

**Authors:** Singtoe Boonrotpong, Sunthorn Sotthibandhu, Chutamas Satasook

**Affiliations:** Department of Biology, Faculty of Science, Prince of Songkla University, Songkhla, Thailand, 90112

**Keywords:** diurnal species, species richness, Scarabaeidae, peninsular Thailand

## Abstract

Species turnover and temporal variation of forest insects were used to explain the ecological succession and ecological segregation between efficiently competing species. In this study, species richness, abundance, and beta-diversity of the genus *Onthophagus* (Coleoptera: Scarabaeidae) assemblages between 2003 and 2007 were described and the diel—flight activity was examined in the disturbed forest and the interior forest of the lowland tropical rain forest at Ton Nga Chang Wildlife Sanctuary in peninsular Thailand. A total of 2,260 individuals of 22 species in 2003 and 2,382 individuals of 24 species in 2007 were collected. Although species richness and abundance did not differ significantly between the two years, all similarity indices were significantly different. The community structure of *Onthophagus* assemblage in 2003 demonstrated a heterogeneous pattern, whereas there was a tendency for the pattern to shift toward a more homogeneous structure in 2007. The temporal variation showed two distinct diel—flight activities; diurnal and crepuscular patterns. Six species were crepuscular (*O. deflexicollis* Lansberge, *O. orientalis* Harold, *O. rudis* Sharp, *O*. sp 1, *O.* sp 2, and *O*. sp 4), whereas most of *Onthophagus* species demonstrated diurnal pattern. Remarkably, five species (*O. taurinus* White, *O. pilularius* Lansberge, *O. punneeae* Masumoto, *O. laevis* Harold, and *O*. sp 3.) could not be classified as either diurnal or crepuscular species. It was suggested that the species turnover was probably influenced by the recovery of the forest structure and the decrease of anthropogenic disturbance. Resource partitioning was suggested to be a key factor for crepuscular adaptation in *Onthophagus* species.

## Introduction

Tropical rain forest is one of the most species rich and functionally important terrestrial ecosystems (Myers et al. 2000). Although the diversity of most arthropod groups in temporal forests is negatively affected by forest modifications and land—use activities, there are few data on arthropods in tropical rain forests despite the importance of these high—diversity systems in research and conservation (Beck and Chey 2007). Dung beetles are an important decomposer involved in nutrient recycling, seed dispersal, and the control of vertebrate parasites; they are therefore an important component of the tropical forest systems (Hanski and Krikken 1991). Local distribution of dung beetles in tropical forests was reported to be strongly influenced by vegetation cover and soil type (Nealis 1977; Doube 1983; Janzen 1983). Moreover, the physical structure of the forest appears to be an important determining factor in the composition and distribution of dung beetle assemblages (Davis and Sutton 1998). Dung beetles can thus be used as an ecological indicator group that reflects the structural differences between habitats. Hence, ecological metrics based on dung beetle assemblages will differ from those of insects that reflect floristic differences through biotope fidelity via plant—feeding specializations (e.g., moths and butterflies). Howden and Nealis (1975) and Klein (1989) employed this useful property of dung beetles to investigate the effects of environmental disturbances on forest diversity and structure.

Although studies on the temporal distribution of insects in tropical forests are scarce (Wolda 1978), the diel activities of dung beetles have been examined in some communities (Jansen 1983; Cambefort 1991; Cambefort and Walter 1991; Doube 1991). Davis (1999) and Feer and Pincebourde (2005) studied the diel flight activity of dung beetles in tropical rain forests of Southeast Asia. They distinguished two main groups of dung beetle assemblage: diurnal and nocturnal guilds. In Borneo, Davis (1999) found a strong overlap in the mean flight activity of the two groups, but more complex temporal patterns require further study within the more species—rich groups. An understanding of diel flight activity is believed to provide an explanation for species packing within the ecosystem.

*Onthophagus* is the most speciose genus of tropical dung beetles and is widely distributed in tropical rain forests, particularly in Southeast Asia (Hanski and Krikken 1991). The distribution pattern and community structure of this genus was influenced by changes of habitat and forest structure (Davis et al. 2001). Consequently, this genus has been selected as a bioindicator in the study of habitat fragmentation and perturbation (Davis et al 2001). It was reported that this genus has dispersed widely from northern to southern Thailand, covering a broad range of habitats in tropical rain forest, and abundantly distributed in the lowland forest at Ton Nga Chang Wildlife Sanctuary of peninsular Thailand (Boonrotpong et al. 2004). Four decades ago, this lowland forest of peninsular Thailand was disturbed by human activity, rubber plantation, logging, hunting, and mining. However, in 1975, the lowland forest was protected from human activity and hence regenerated. As a consequence, the area affected by rubber plantation was left undisturbed for about 20 years (Sawangchote 1998). In 2003, the structure of this lowland forest was comprised of fragmented patches of vegetation, and was still disturbed by human activity and rubber plantation; it was secondary forest with trees more widely spaced than in primary forest. By 2007, the forest structure had gradually changed and was characterized by a full ceiling canopy and several layers of understory (pers. obs.), showing that forest regeneration reflected species turnover of the dominant species of *Onthophagus*.

In this study, the genus *Onthophagus* in the lowland forest of peninsular Thailand was collected and monitored in 2003 and 2007. The aims of the study were to compare species composition and to determine factors influencing species turnover of *Onthophagus* species from 2003 to 2007, and to study the variation in flight activity within the *Onthophagus* species.

## Materials and Methods

### Study area

The study area was located in Ton Nga Chang Wildlife Sanctuary, Songkhla Province, peninsular Thailand, situated on the Banthat mountain range (15° 33′ to 16° 23′ N, 98° 33′ to 99° 07′ E). Ton Nga Chang Wildlife Sanctuary is one of the important tropical rain forests in peninsular Thailand. It comprises two forest types: lowland forest and dry forest. The lowland forest is situated on high steep limestone approximately 300 m a.s.l., whereas the dry forest is located at 300–500 m a.s.l. The forest characteristics are affected greatly by both northeastern and southwestern monsoons. The dominant trees in the lowland forest included the families Euphorbiaceae, Annonaceae, Dipterocarpaceae, Lauraceae and Meliaceae, and the canopies covered approximately 70% of the area (Sawangchote 1998). The study site was selected in the lowland forest where there was a continuity of disturbed forest and interior forest. A total distance of 1000 m was included in the study (500 m each for disturbed forest and interior forest).

In 2003, the dominant species in this study site were from the families Euphorbiaceae, Annonaceae, Dipterocarpaceae, Lauraceae, and Meliaceae (Boonrotpong et al. 2004). Although the forest structure in 2007 was observed to be different from that of 2003, the dominant species were still from the families Euphorbiaceae, Annonaceae, Dipterocarpaceae, Lauraceae, and Meliaceae (pers. obs.).

### Sampling methods

Dung beetles in the genus *Onthophagus* (Coleoptera: Scarabaeidae) were sampled in 2003 and 2007 in the lowland forest of Ton Nga Chang Wildlife Sanctuary. Dung beetles were collected bimonthly in both rainy and dry seasons during February 2003 to March 2004, and February 2007 to March 2008. Two line transects were set up in parallel at 200 m apart. Each line transect extended approximately 1000 m from the disturbed forest (500 m) to the interior forest (500 m). The collecting sites within the line transect reflected the gradient of forest community along the distance from disturbed forest to interior forest. Along each line transect 20 baited—pitfall traps were set at 50 m apart to avoid pseudo—replication. Of those, 10 traps were placed in the disturbed forest (sampling sites: S1–S10 for 2003, and S21–S30 for 2007), and another 10 were placed in the interior forest (sampling sites: S11–S20 for 2003, and S31–S40 for 2007). Each trap was baited with 400 g of fresh pig dung (Hanboonsong 1998; Boonrotpong et al. 2004). The baited dung was put in a plastic container (12 × 12 × 10 cm), which was covered with a plastic roof for protection from sun and rain. Dung beetles were collected and the traps were re—baited with fresh pig dung every 24 hours (on average) for two consecutive days. Trapped specimens were preserved in 95% ethanol. *Onthophagus* species were identified to species level using Paulian (1945), Balthazar (1963), and Zunino (1975, 1979a, 1979b, 1981).

The environmental variables of light intensity, vegetation cover, and temperature were measured at each collecting site for each study visit. Light intensity was measured as an average percentage of light intensity on the forest floor, and the percentage cover of vegetation in each quadrat at each sampling site (1 × 1 meter) represented vegetation cover. Temperature was measured using regularly calibrated meters.

For the diel flight activity study, 30 baited—pitfall traps were randomly placed in the lowland forest. Dung beetles were collected and the traps were re—baited at three—hour intervals over a 24 hour period. The trapped specimens were separated in each collecting time and were preserved in 95% ethanol. *Onthophagus* species were identified to species level using Paulian (1945), Balthazar (1963), and Zunino (1975, 1979a, 1979b, 1981).

All of the voucher specimens for each sampling method were deposited in the insect collections of the Princess Maha Chakri Sirindhorn Natural History Museum at Prince of Songkla University, Thailand.

### Data analysis

The species richness was used to describe the number of different species encountered within each habitat in each year. Species richness was estimated using Fisher's alpha, which was chosen for the comparison of diversity indices since the method is appropriate for data assessed from a large sample size (in this study, n = 320) and in which abundance of species follows a log series distribution (Magurran 2004). True species richness in each year was examined from the observed number of species using Estimates version 7.0 software (Colwell and Coddington 1994). The effect of temporal variation (2003, 2007) in species richness and abundance of *Onthophagus* species was examined by one—way ANOVA.

Two methods of analysis were used to calculate the beta—diversity. The first was the Bray—Curtis index to calculate species similarity using the number of individuals and number of species collected in the two study years:
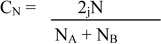

where N_A_ is the total number of individuals in 2003, N_B_ is the total number of individuals in year 2007, 2_j_N is the sum of the lower of the two abundances of each one of the species found in both years. The Bray—Curtis value ranges from 1 (when the two samples are identical) to 0 (when there are no shared species). This index is appropriate for calculating species turnover because it reflects the difference in absolute abundance rather than in relative abundance (Magurran 2004). For the second method, Whittaker β_w_, Cody β_c_, Routledge βi, Routledge β_e_, and Wilson and Shmida β_f_ (Vellend 2001) were used to measure the species turnover of *Onthophagus* assemblage by calculating species composition between 2003 and 2007.

Correlations between relative abundance of each species and environmental factors were investigated at each site and year by canonical correspondence analysis (CCA), incorporating the unimodal response of species to environmental gradient. The CCA produced an ordination diagram in which species composition and sampling site were represented by points and environmental factors by vectors. The significance of the relationship between sets of species composition and environmental variables was calculated by Monte Carlo permutation test (999 runs) using PC—ORD program version 3.2.

Multivariate analysis of variance was further conducted between species richness of the *Onthophagus* and the potential physical factors that may be responsible for changes in dung beetle composition.

The diel—flight activity for each species was examined using multiple dimensional scaling (MDS) based on Bray—Curtis similarity in order to classify flight behavior. For each species, the average number of individuals (collected every three hours) was calculated from the combined data of 2003 and 2007. The numbers were plotted against time over a 24—hour period to identify the activity rhythm of *Onthophagus* assemblage.

## Results

### Species composition

**Figure 1.**
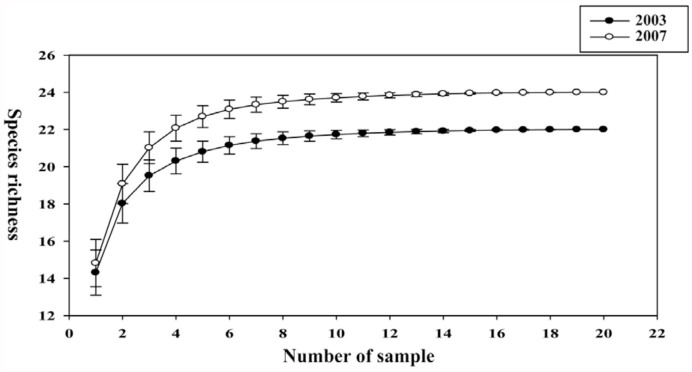
Species accumulation curve of *Onthophagus* species across 2003 and 2007. High quality figures are available online.

Species accumulation curves showed asymptotic patterns, indicating complete collection of *Onthophagus* for estimating species richness in each year (Figure 1). Although the number of species and number of individuals were similar between 2003 and 2007, true species richness of *Onthophagus* was higher in 2007 than 2003 (Figure 1).

**Table 1.**
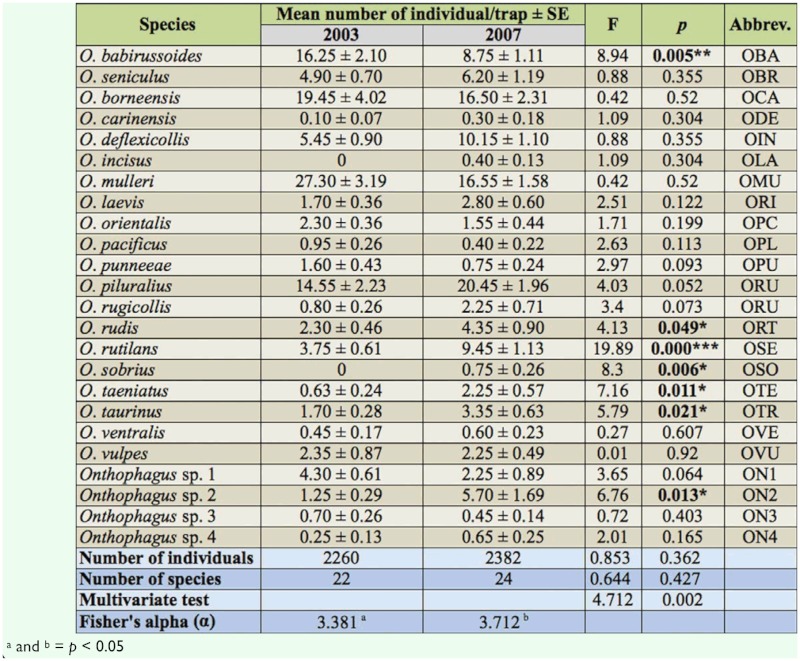
Mean number of individuals per trap of *Onthophagus* recorded in 2003 and 2007 and species diversity.

A total of 2260 *Onthophagus* representing 22 species were found in 2003, whereas total of 2382 *Onthophagus* belonging to 24 species were sampled in 2007 (Table 1). Two species (*Onthophagus laevis* Harold and *O. sobrius* Balthasar) were new invaders to the area and were collected exclusively in 2007. Considering the numbers of individuals per trap (10+) in 2003, there were four dominant species: *O. mulleri* Lansberge had the highest number (27.30 ± 3.19), followed by *O. carinensis* Boucomont (19.45 ± 4.02), *O. babirussoides* Krikken and Huijbregts, MS (16.25 ± 2.10), and *O. rugicollis* Harold (14.55 ± 2.23) (Table 1). In 2007, there were four dominant species: *O. rugicollis* showed the highest number of individuals per trap (20.45 ± 1.96), followed by *O. mulleri* (16.55 ± 1.58) and *O. carinensis* (16.50 ± 2.31), and *O. incisus* Harold (10.15 ± 1.10). The mean numbers of individuals per trap of *O. babirussoides, O. seniculus* Fabricius, *O. rutilans* Sharp, *O. sobrius, O. taeniatus* Boucomont, *O. taurinus* White, and *O*. sp 2. differed significantly between 2003 and 2007 (Table 1).

The results in Table 1 demonstrate that Fisher's alpha (α) diversity index of *Onthophagus* species was significantly higher in 2007 than in 2003 (α2007 (3.712), α2003 (3.381), *p* < 0.05). Numbers of species and abundance were not significantly different between 2003 and 2007 (species richness, *F*_1,39_ = 0.644, *p* = 0.427; number of individuals, *F*_1,39_ = 0.853, *p* = 0.362) (Table 1).

### Species turnover

The calculated Bray—Curtis index indicated 56.47% similarity between the years 2003 and 2007. In 2003, the similarity of *Onthophagus* species across collection time was 60.41%, whereas in 2007 it was 62.23%. All betadiversity indices differed significantly between 2003 and 2007 (Routledge βe = 1.0296, *p* < 0.05; Cody β_c_ = 1, p < 0.05; Wilson and Shmida β_t_ = 0.4345, *p* < 0.05; Whittaker β_w_ = 0.0435, *p* < 0.01; and Routledge β_e_ = 0.0292, *p* < 0.01) (Table 2).

**Figure 2.**
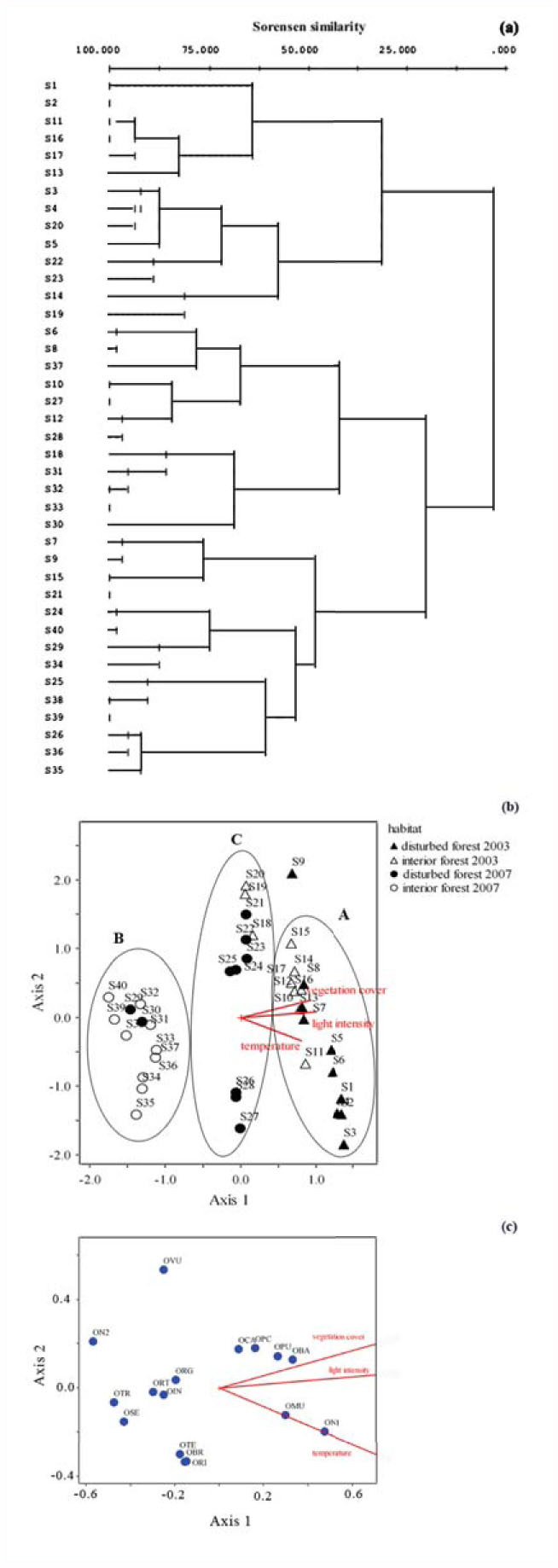
Ordination diagram from Canonical Correspondence Analysis (CCA): a) Single-link diagram (Sorensen similarity) of sampling site in 2003 and 2007, b) Canonical diagram between sampling site of Onthophagus species assemblage and environmental factors, c) Ordination diagram (biplot) from CCA between Onthophagus species and environmental factors. High quality figures are available online.

The result from cluster analysis by CCA ordination in the sampling sites across the two years (S1–S40) based on dissimilarity of species compositions (Figure 2a) demonstrated that the distribution pattern of *Onthophagus* assemblage was divided into two main groups at 100% dissimilarity: Clade A and Clade B. Clade A included 12 sampling sites from disturbed and interior forests of 2003 (S1, S2, S3, S4, S5, S11, S13, S14, S16, S17, S19, S20), and two sampling sites from disturbed forest in 2007 (S22, S23). This clade was further separated into two subclades at 70% dissimilarity. By contrast, Clade B consisted of 26 sites, of which 18 sites were in the disturbed and interior forests of 2007 (S21, S24, S25, S26, S27, S28, S29, S30, S31, S32, S33, S34, S35, S36, S37, S38, S39, S40), whereas the remaining eight sites belonged to the disturbed forest of 2003 (S6, S7, S8, S9, S10, S12, S15, S18).

Figure 2b showed the correlation between dung beetle composition in each sampling site and the environmental factors (light intensity, vegetation cover, and temperature). The result from canonical ordination analysis (axis 1, eigenvalue = 0.108, Monte Carlo permutation, *p* < 0.01; axis 2, eigenvalue = 0.067, Monte Carlo permutation, *p* < 0.01) divided dung beetle composition in each study site into three different correlation groups (A, B, C). Group A included dung beetle assemblages in the sampling sites of 2003 and demonstrated a positive correlation with all environmental factors. It showed that seven sampling sites (S1–S6, S11) were strongly affected by temperature, whereas vegetation cover and light intensity had more influence on the remaining sites (S7, S8, S10, S12, S13, S14, S15, S16, S17). Group B included 12 sampling sites (S29–S40) that were negatively influenced by three environmental factors. However, 11 sampling sites in group C showed no correlation with any environmental parameter.

**Table 2.**
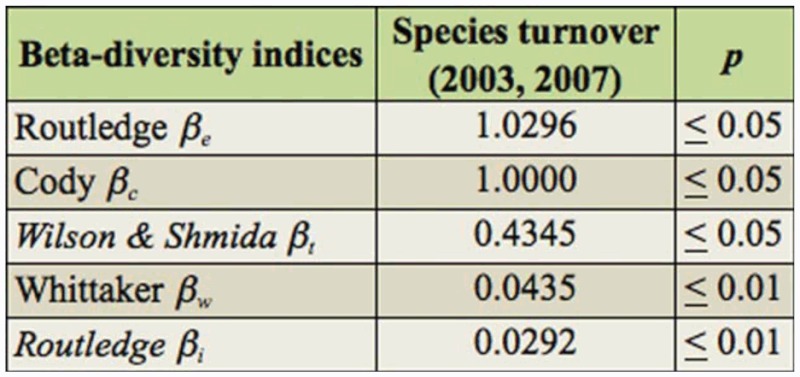
Beta—diversity indices measuring species turnover of *Onthophagus* assemblage between 2003 and 2007.

The correlation between *Onthophagus* species and environmental factors is demonstrated in Figure 2c. Light intensity and vegetation cover were correlated positively with the distribution of four *Onthophagus* species (*O. carinensis*, *O. pacificus* Lansberge, *O. punneeae* Masumoto, *and O. babirussoides*), whereas *O. mulleri* and *O.* sp.1 were strongly influenced by temperature. However, *O. incisus, O. rutilans, O. rugicollis, O. taurinus*, and *O. seniculus* showed a negative correlation with environmental factors.

### Diel flight activity

**Figure 3.**
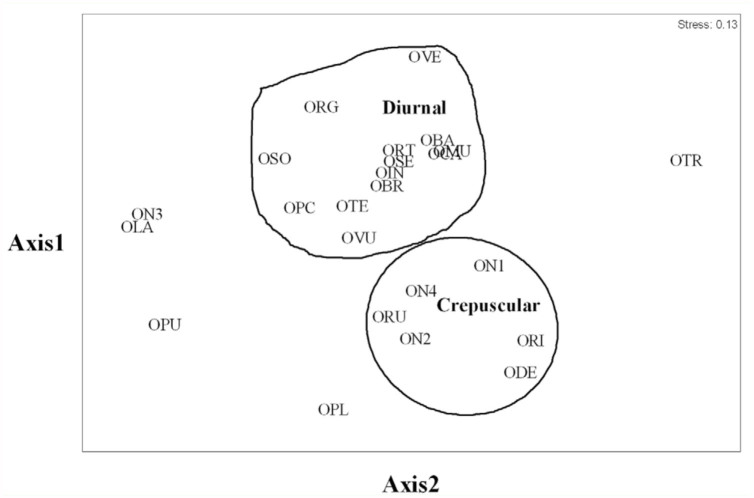
Multiple dimensional scaling (MDS) of diel flight activity of *Onthophagus* species based on the similarity coefficient. High quality figures are available online.

**Figure 4.**
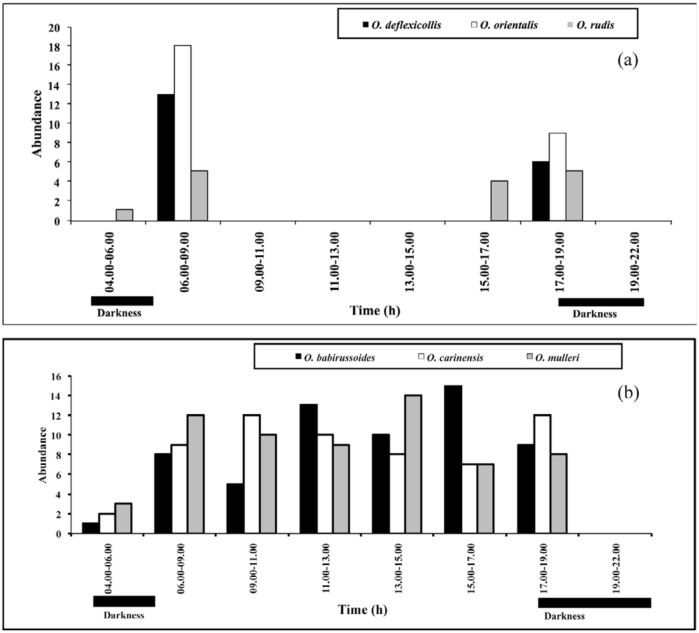
Diel flight activity of crepuscular and diurnal species: (a) crepuscular species, *Onthophagus deflexicollis, O. orientalis, O. rudis* and (b) diurnal species, *O. babirussoides, O. carinensis* and *O. mulleri*. High quality figures are available online.

The result of similarity coefficient using multiple—dimensional scaling (MDS) showed that *Onthophagus* assemblages were divided into two main groups; diurnal and crepuscular species (stress = 0.13) (Figure 3). Six species were defined as crepuscular species; *O. deflexicollis* Lansberge, *O. orientalis* Harold, *O. rudis* Sharp, *O.* sp. 1, *O*. sp. 2, and *O*. sp. 4, whereas 13 *Onthophagus* species were diurnal species (Figure 3) and five species (*O. laevis, O. piluralius, O. punneeae, O. taurinus*, and *O*. sp. 3) could not be defined as either diurnal or crepuscular species. Figure 4 shows that the crepuscular species (*O. deflexicollis, O. orientalis, O. rudis*) were active during dawn (04:00–08:00) and dusk (17:00–19:00), whereas the diurnal species (*O. babirussoides, O. carinensis* and *O. mulleri*) flew preferentially during the day.

## Discussion

### Species composition and turnover

Although number of species and abundance of *Onthophagus* in this study did not differ significantly between 2003 (22 species) and 2007 (24 species), different analyses revealed that the ensemble composition of *Onthophagus* species changed from 2003 to 2007. In this study, seven species (*O. babirussoides, O. seniculus, O. rudis, O. rutilans, O. sobrius, O. taeniatus*, and *O. taurinus*) differed significantly between 2003 and 2007. *Onthophagus babirussoides* was a dominant species in 2003 but not in 2007. This species has been reported as a common species in disturbed forest and prefers a dry, low humidity and shallow soil type habitat (Hanski and Cambefort 1991), similar to our finding in the disturbed forest in 2003. In 2007, evidence of new invaders (*O. laevis* and *O. sobrius*) was recorded. Hanboonsong et al. (1999) suggested that *O. laevis* and *O. sobrius* prefer a highly humid and deep soil type, which was characteristic of our forest study sites in 2007.

The CCA diagram plotting between sampling sites and environmental factors (Figure 2) demonstrated that habitat recovery played an important role in regulating species composition and turnover of *Onthophagus* species. In 2003, ensemble composition of *Onthophagus* showed a distinct heterogeneous pattern. The cluster analysis by CCA ordination clearly indicated that distribution of species composition in 2003 separated into two distinct groups (12 sampling sites in Clade A, eight sampling sites in Clade B, Figure 2a). The result suggested a probable heterogeneous pattern within sampling sites. In contrast, 18 of 20 study sites in 2007 were grouped together in Clade B, suggesting that the ensemble composition of *Onthophagus* had a more homogeneous pattern within the study plots.

The results indicated that the composition of *Onthophagus* species may have gradually shifted from a heterogeneous pattern to a homogeneous pattern over time. The probable factor explaining the difference in *Onthophagus* ensemble was assumed to be the recovery of forest structure and community. This hypothesis was confirmed by the result of the multivariate analysis of variance comparing forest structure (site, vegetation cover, temperature, and light intensity) and species composition collected along the study site (*F* = 4.712, *p* < 0.01, Table 1). Additional results from CCA analysis (Figure 2b) indicated that the association of species composition in each study site with environmental factors was divided into three distinct groups: group A included species composition in the sites that correlated positively with light intensity, vegetation cover, and temperature. The forests with high light intensity, high temperature, and dense vegetation cover were mostly disturbed forests, which consisted of a widely spaced canopy that allowed sunlight to reach the forest floor, and contained more undergrowth or small trees. Moreover, forest—gap formation provides the opportunity for microhabitat differentiation and several microclimate variations in disturbed forest, and probably provides suitable opportunities for species diversification and abundance in the area (Spitzer et al. 1997). All of the study sites (16 sites) in group A were coincidentally the sites in the forest of 2003. This clearly indicated that the forest structure in 2003 was mainly disturbed forest, and the composition of *Onthophagus* species was affected positively by the three factors. By contrast, group B included sites where species composition was negatively affected by light intensity, vegetation cover, and temperature. The result suggested that the forest structure of the sites in group B was different from the structure of those in group A. The CCA analysis clearly revealed that all sampling sites (12 sites) in group B were in the forest of 2007. It was recorded that in 2007, the forest structure was characterized by a full ceiling canopy, but there were still a few layers of understory. The area was 50% covered by closed canopy, which prevented penetration of sunlight to the ground; hence, the ground floor included large trees with little undergrowth and few small trees. However, the dominant plant species in both 2003 and 2007 were the same, from the families Euphorbiaceae, Annonaceae, and Dipterocarpaceae (Sawangchote 1998). Group C consisted of 11 sites that demonstrated no correlation with environmental parameters; these sites were considered to be in a transition zone. Of those, eight sites were the previous sites in 2003 (S21–S28, equivalent to S1–S8). This suggests that S1–S8 were probably highly disturbed in 2003 and slowly recovered by 2007. The sites in this transition zone may shift over time toward group B if there is no further disturbance. The transition zone was observed to be an open forest that consisted of more undergrowth, medium trees, and secondary canopy. Family Poaceae was the main vegetation cover and was the dominant species in this forest zone.

The CCA analysis (Figure 2c) further suggested that the distribution of four *Onthophagus* species (*O. carinensis, O. paciflcus, O. punneeae*, and *O. babirussooides*) was regulated by high light intensity and dense vegetation cover. This study was consistent with the suggestion by Davis et al. (2001) that these species favor an open area to build up nesting and breeding sites. Whereas *O. mulleri* and *O.* sp.1 were strongly influenced by temperature, these species were reported to be specialist species in disturbed areas (Davis et al. 2001; Boonrotpong et al. 2004). However, *O. incisus, O. rutilans, O. rugicollis, O. seniculus*, and *O. taurinus* showed a negative correlation with environmental factors. The result was similar to the finding of Shahabuddin et al. (2005), who reported that *O. incisus, O. rutilans*, and *O. rugicollis* prefer nesting and breeding sites with low light intensity and low temperature.

Boonrotpong et al. (2004) reported that mammal diversity and dung type were different in the three forest areas. The diversity of mammals in the disturbed forest, especially the herbivores, was lower than in the interior forest. Hence, dung availability and dung quality may have been scarce, and consequently there were fewer *Onthophagus* species in 2003. This is supported by Hanski and Cambefort (1991), who suggested that *Onthophagus* species favored herbivorous mammal droppings. By 2007, as a result of forest recovery, succession, and non—disturbance by human activities, the forest structure and plant diversity of the disturbed forest had gradually recovered from a disturbed habitat to secondary forest. For this reason, some large herbivorous mammals may radiate, since the secondary lowland forest could provide nesting and breeding sites. During the field survey in 2007, more dung droppings of large herbivorous mammals were observed than in 2003 (pers. obs.). Large herbivorous mammals play an important role in the diversity and distribution of dung beetles in the tropical rain forest (Estrada et al. 1993; Davis et al. 2001; Boonrotpong et al. 2004). In addition, difference in food resources provided a good explanation for the availability of consumer communities (Strong et al. 1984). Thus, ensemble composition of *Onthophagus* showed a tendency to move toward a homogeneous pattern in 2007. Our conclusions are similar to those of Doube (1991) and Hanski and Cambefort (1991), who suggested that the diversity of dung availability, distribution, and quality, and changes of vegetation structure were the major factors for the regulation of biodiversity of dung beetles in savannas and tropical rain forests. However, these factors are not necessarily independent but may have additive effects on dung beetle ensembles (Shahabuddin et al. 2005).

Although the number of species and the abundance of *Onthophagus* were not significantly different between 2003 and 2007, ensemble composition and species turnover of *Onthophagus* gradually changed from a heterogeneous to a homogeneous pattern.

### Diel flight activity

Although most *Onthophagus* species showed diurnal flight pattern (13 species), a few species were recorded as crepuscular (six species), and five species demonstrated both diurnal and crepuscular behavior. Hanski and Krikken (1991) studied dung beetles in tropical rain forests in Southeast Asia and suggested that *Onthophagus* species are diurnal. Davis (2001) investigated dung beetles in Borneo and reported a similar result. Our finding differs from those of previous reports. It was suggested that intra—and inter—specific competition occurs for dung dropping and time of feeding among the *Onthophagus* species, and as a result, crepuscular behavior was adopted to avoid competition. Hanski and Krikken (1991) proposed the term “minor habitat” for animal dropping, and defined it as a “patchy and ephemeral” resource. Dung beetles in the genus *Onthophagus* were recognized as good competitors with high potential to reach and exploit food resource in a short period of time. After the fresh dropping was consumed by the first batch of dung invaders it rapidly reduced its attractiveness to other beetles (Hanski and Cambefort 1991), leading to an intense competition for food resources. Resource partitioning may therefore provide a key to successful coexistence of species using the same resources. Although there was no obvious evidence for resource partitioning in this study, flight period may represent an indirect indicator for resource partitioning.

The results from this study revealed two peaks of flight activity in the crepuscular species at 06:00–09:00 and 17:00–19:00 (Figure 4a), suggesting an adaptation to the periods of mammalian defecation. In our study site, wild boar (*Sus scrofa*) is an important provider of dung dropping (Boonrotpong et al. 2004). Ickes (2001) reported that the preferred defecation period of this wild mammal is early in the morning and during late afternoon. Caveny et al. (1995) reported a similar result that resource partitioning plays a major role in the regulation of the pattern of flight activity in *Onitis* species.

In conclusion, the disturbed forest may have gradually recovered and changed to secondary forest and, in time, will become primary forest. Habitat succession and rehabilitation play an important role in determining tropical rain forest dung beetle assemblages. Resource partitioning was suggested to be a key factor for crepuscular behavior in *Onthophagus* species.
